# Neuroinflammation, memory, and depression: new approaches to hippocampal neurogenesis

**DOI:** 10.1186/s12974-023-02964-x

**Published:** 2023-11-27

**Authors:** Anbiao Wu, Jiyan Zhang

**Affiliations:** Beijing Institute of Basic Medical Sciences, Beijing, 100850 China

**Keywords:** Major depressive disorder, Neuroinflammation, Memory impairment, Hippocampal neurogenesis, Neurotrophic factors

## Abstract

As one of most common and severe mental disorders, major depressive disorder (MDD) significantly increases the risks of premature death and other medical conditions for patients. Neuroinflammation is the abnormal immune response in the brain, and its correlation with MDD is receiving increasing attention. Neuroinflammation has been reported to be involved in MDD through distinct neurobiological mechanisms, among which the dysregulation of neurogenesis in the dentate gyrus (DG) of the hippocampus (HPC) is receiving increasing attention. The DG of the hippocampus is one of two niches for neurogenesis in the adult mammalian brain, and neurotrophic factors are fundamental regulators of this neurogenesis process. The reported cell types involved in mediating neuroinflammation include microglia, astrocytes, oligodendrocytes, meningeal leukocytes, and peripheral immune cells which selectively penetrate the blood–brain barrier and infiltrate into inflammatory regions. This review summarizes the functions of the hippocampus affected by neuroinflammation during MDD progression and the corresponding influences on the memory of MDD patients and model animals.

**Supplementary Information:**

The online version contains supplementary material available at 10.1186/s12974-023-02964-x.

## Introduction

Characterized by depressed mood, persistent anxiety, loss of interest in daily activities, and even suicidal thoughts, major depressive disorder (MDD) has been ranked as the third leading cause of the disease burden worldwide, especially for the elderly population, with a reported average incidence of 13.3% in 2021 [1]. MDD stands out among psychiatric disorders for that patients are at significantly increased risk of premature death by suicide and other medical conditions, such as sleep disorders or cardiovascular diseases [2, 3]. The neurobiology of MDD can be categorized into three major aspects: the reduced serotonin neurotransmission, the dysfunctional hypothalamus–pituitary–adrenal (HPA) axis, and the impaired neurogenesis in the dentate gyrus of the adult hippocampus [4–6]. To further explore the molecular and cellular mechanisms of MDD development and potential treatment, several types of animal models have been established and are generally recognized as sufficient to mimic the behavioral characteristics of MDD patients, including learned helplessness (LH), chronic unpredictable mild stress (CUMS), and repeated social defeat (RSD) [7–10].

In both MDD animal models and patients, various types of cognitive deficits have been widely observed, such as attenuated memory performance [11, 12]. Short-term and long-term memory have been manifested to involve different neural systems [13]. The well-studied case of Henry Molaison, in which the patient showed memory impairment after the bilateral removal of the hippocampus, pioneered the exploration of memory formation by providing the first clear correlation between memory and the hippocampus [14]. To date, the structures of the medial temporal lobe, including the hippocampus, have been verified to be responsible for episodic long-term memory processing [15] and defined as the medial temporal lobe memory system [16]. Therefore, the conditions of the hippocampus during MDD progression require our attention.

Initially referred to as the inflammatory functions of microglia under pathological conditions, the definition “neuroinflammation” is now used ubiquitously to describe all immunological activities within the central nervous system (CNS) toward acute and chronic diseases, including traumatic, infectious, ischemic, autoimmune, and degenerative ones [17]. The previous theory of “brain immune privilege”, which postulates that the CNS could tolerate the introduction of antigens and thus limit any excessive inflammatory damage, was based on the absence of any lymphatic drainage and the blood–brain barrier (BBB) [18]. However, recent discoveries have improved the general understanding of immune surveillance of the CNS and remotivated discussions about the soundness of “brain immune privilege” [19]. In healthy brains, various types of lymphocytes and myeloid cells have been found to be located in the meninges, cerebrospinal fluid (CSF), and parenchyma [20, 21]. The current perception of neuroinflammation includes three major categories: microglia and resident macrophages in the CNS mediate innate immune responses [22], more peripheral immune cells migrate and infiltrate into the CNS during neuroinflammation [23], and infiltrated T cells may play an important role in adaptive immunity [24–26].

The relationships between neuroinflammation and neurodegenerative disorders have been gradually elucidated [27, 28]. In specific, multiple psychosocial stressors have been demonstrated to accelerate the development of neuroinflammation and mental illness according to the clinical evidence [29]. Ongoing neuroinflammation could in turn induce depressive-like behaviors [30, 31] or promote MDD progression [32]. Currently available antidepressants eliminate depressive symptoms for only 37% of MDD patients [33], together with an overall cumulative remission rate of 67% [33]. These facts suggest the potential advantages of considering immune-regulatory mechanisms in the development of MDD treatment, compared to the current first-line antidepressants and their focus on inhibiting neurotransmitter reuptake or sensitizing their receptors. Moreover, the abilities of these antidepressants to ameliorate cognitive deficits in patients suffering from MDD, especially memory impairments, have not been evaluated sufficiently during their development [34]. Since the neuroinflammatory dynamics observed in MDD animal models have been reported to regulate memory impairments [35, 36] and hippocampal neurogenesis [37, 38], this review intended to summarize the links and interactions between neuroinflammation and cognitive deficits which are correlated with the proliferation and functions of neurons in the hippocampus.

## The effects of neuroinflammation on memory impairment via the modulation of hippocampal neurogenesis

### Neurogenesis in the hippocampus

Among the different regions in the cerebral parenchyma, the hippocampus is predominantly associated with memory functions. Anatomically, the mammalian hippocampus consists of the dentate gyrus (DG), subiculum, and cornu ammonis (CA) areas 1–3 [39]. Neurogenesis is the process by which neural stem cells (NSCs) gradually differentiate into mature neurons [40]. Neurotrophins (NTs) serve as the essential and irreplaceable mediators during this process [41]. The DG is one of two recognized microenvironments/niches for neurogenesis in adult mammalian brains, which refers to the sub-granular zone (SGZ) of the DG and the border of the lateral ventricles of the brain (sub-ventricular zone, SVZ) [42]. The DG can be divided into three distinct layers: the molecular layer (ML) containing mainly the dendritic projections of granule cells (GCs), the granular cell layer (GCL) containing the cell bodies of GCs, and the polymorphic layer named the hilus [43]. The thin layer of cells forming the border of both the GCL and hilus is defined as the SGZ, where NSCs are located together with other cell types, such as neural progenitor cells (NPCs), GCs, interneurons, mossy cells, astrocytes, oligodendrocytes, and microglia [44, 45]. Activated NSCs in the DG differentiate into oligodendrocytes, astrocytes, and mature neurons through distinct differentiation pathways [46]. The proliferation, differentiation, and survival of these NSCs are mediated by various factors within the SGZ microenvironment [47], such as neurotransmitters, growth factors, cytokines, and hormones secreted from other cell types [48, 49].

The prolonged capacity of NSCs in the SGZ to self-renew, retain cellular identity, and react to specific signals is fundamental for mammals to ensure life-long neurogenesis in the DG [50]. The differentiation from NSCs into mature neurons includes a series of sequential and time-consuming processes [51]. Type 1 NSCs, also named radial glial cells for their radial morphology, are mainly located in the SGZ [52]. Newborn type 1 NSCs first transdifferentiate into transient amplifying progenitor cells (TAPCs), which exhibit limited self-renewal ability and a higher proliferation rate than NSCs and can be further divided into subtypes 2a and 2b [53]. Type 2b TAPCs continue to differentiate to generate neuroblasts (NBs)/type 3 cells and migrate to the inner half of the GCL [54]. The process from type 1 NSCs to type 3 NBs normally lasts 1–4 days, during which 60% of newborn NPCs undergo cell death by apoptosis [55]. Type 3 NBs go through another 5–27 days of differentiation into immature neurons [56] and subsequently 28–60 days into mature neurons with integrated synaptic structure [57]. That is, the entire process of neurogenesis in the adult mammalian brains takes 5–11 weeks after the birth of NSCs, which ensures the persistent maturation of fully functional neurons throughout the life circle [58]. Therefore, developments in techniques and equipment have been continually focusing on evaluating neurogenesis more precisely for both animal models [59] and human patients [60].

### Neuroinflammation inhibits hippocampal neurogenesis during depression

In both animal models and human patients, the participation of neuroinflammation during MDD progression has been increasingly recognized. For example, Haapakoski et al. [61] discovered strong correlations between depression and the upregulation of peripheral markers of inflammation in CSF samples, such as interleukin-6 (IL-6) and C-reactive protein (CRP). In addition, interferon-α (IFN-α) in the brain parenchyma has been reported by Su et al. to be interrelated with the pathogenesis of MDD [62]. In a meta-analysis, the clinical use of various anti-inflammatory drugs, including nonsteroidal anti-inflammatory drugs (NSAIDs), cytokine inhibitors, statins, and minocycline, significantly ameliorated depressive symptoms in some MDD patients [63]. In mice with depressive-like behaviors, elevated transcription of proinflammatory and injury-repair genes was observed in hippocampal tissues [64].

**Fig. 1:**
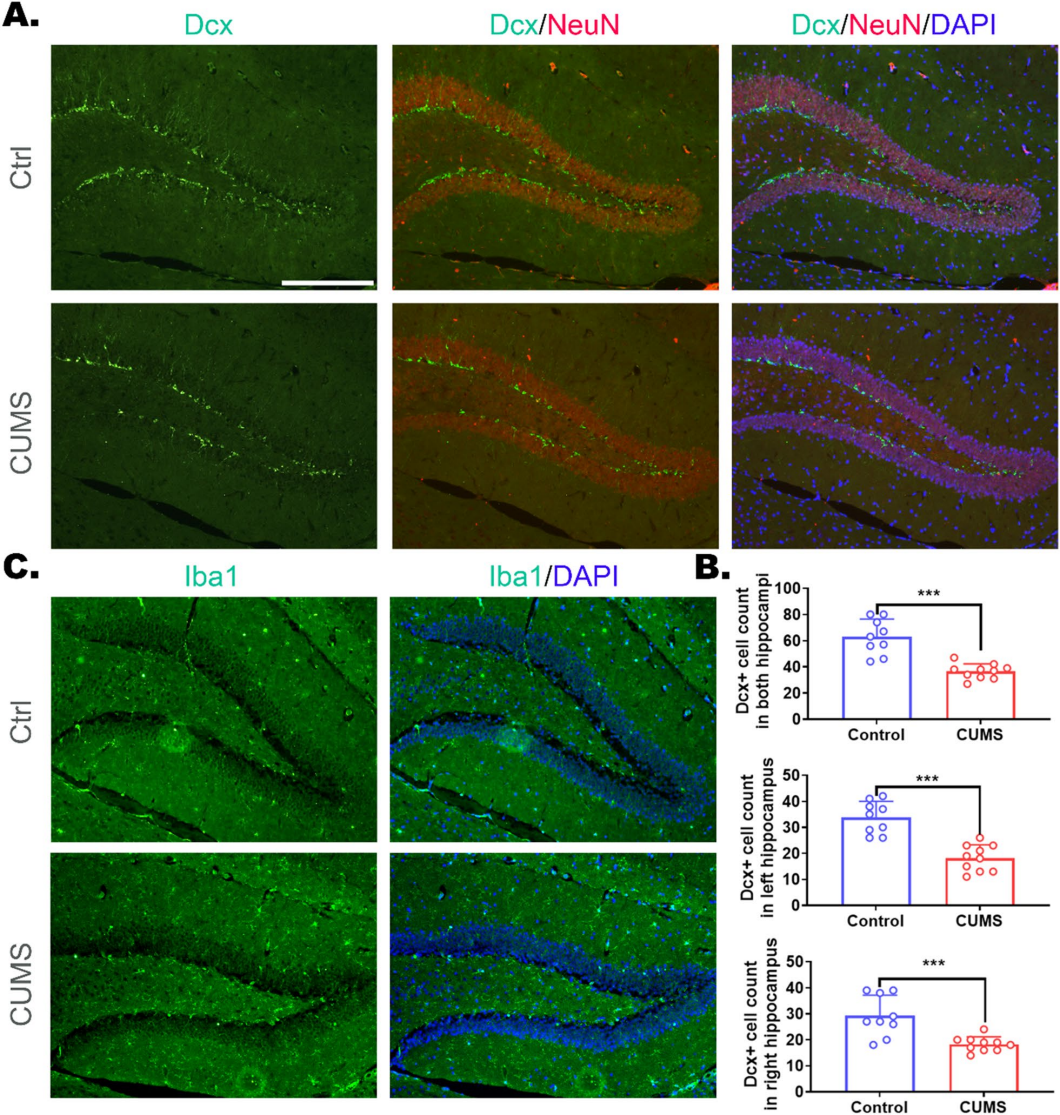
Microglial activation and attenuated hippocampal neurogenesis in DG were observed in depression model of mice. **A** Immunostaining with anti-Dcx and anti-NeuN of hippocampal coronal sections of mice under CUMS treatment. **B** The statistical analysis of Dcx^+^ cells located at the SGZ. The mice received CUMS treatment showed significantly decreased newborn, immature neurons as indicated by anti-Dcx staining (***p < 0.001 vs. Ctrl). **C** Immunostaining with anti-Iba1 of hippocampal coronal sections of mice under CUMS treatment. The mice received CUMS treatment showed increased Iba1^+^ signals in the DG, which might suggest increased numbers of reactive microglia. The experimental details are described in Additional file 1

MDD-induced neuroinflammation has been reported to affect cognitive and memory functions by affecting neurogenesis in the DG (Fig. 1A, B; the corresponding experimental methods were described in Additional file 1), leading to inattention, impaired working memory, and increased negative cognitive bias [65, 66]. Mckim et al. [67] reported that RSD promoted neuroinflammation (mainly through microglial modulation) in the mouse hippocampus, resulting in transient spatial memory impairment. Although the cell number and proliferation rate of NPCs were not affected by RSD, their differentiation into mature neurons was significantly inhibited. Correspondingly, minocycline, an inhibitor of microglial differentiation into proinflammatory phenotype, was found to penetrate the BBB into the CNS and suppress the recruitment and infiltration of peripheral lymphocytes into the hippocampus during RSD [68], thereby attenuating spatial memory impairment after RSD by inhibiting neuroinflammation [36].

Previous studies have shown that exercise serves as a powerful inducer of hippocampal neurogenesis, and the benefits of regular exercise on cognitive function might be attributed to its proneurogenic effects [69]. In addition, in the DG with improved neurogenesis, newborn mature neurons also exhibit enhanced synaptic plasticity [70] and long-term potentiation (LTP) [71]. In 1999, Gage et al. found [72] that voluntary running increased the proliferation and survival of new neurons in the mouse DG. In subsequent studies, it was further demonstrated that long-term voluntary running in mice not only augmented hippocampal neurogenesis, but also enhanced the spatial learning ability of mice [73]. This exercise-induced hippocampal neurogenesis has been shown to be necessary to improve spatial memory functions in rodents [74] and to be limited to the hippocampus, which means that neurogenesis in the SVZ is not affected [75]. In addition, exercise-induced hippocampal neurogenesis is accompanied by increased LTP, dendritic spine density, and reactivity to NTs, together with increased fibroblast growth factor-2 (FGF-2) and vascular endothelial growth factor (VEGF) expression levels [76, 77]. The interplay between exercise and cognitive functions influenced by neuroinflammation, however, was investigated much later. Wu et al. [78] demonstrated that lipopolysaccharide (LPS)-induced neurogenesis deficits and impaired Morris water maze (MWM) test performance in mouse models could be gradually rescued during the subsequent 5 weeks of forced treadmill training. Nevertheless, negative results were also observed in subsequent studies [79], which was explained by the authors’ hypothesis that exercise interventions might miss the time window of the influence of neuroinflammation on neurogenesis. For human subjects, similar effects of exercise on regulating neuroinflammation, promoting cognitive functions, and maintaining the structural integrities of the corresponding brain regions have also been demonstrated, but the existing evidence is not sufficiently informative as research at the animal level and needs to be further studied [80, 81].

### Neurotrophins and hippocampal neurogenesis

#### Neurotrophin definition and functions in neurogenesis

The understanding of NTs started from the discovery of nerve growth factor (NGF) [82]. NTs are growth factors that play irreplaceable roles in neuronal proliferation, survival, development, function, and plasticity [83]. To date, the discovered NTs could be listed as follows: NGF, brain-derived neurotrophic factor (BDNF), neurotrophin-3/4 (NT-3/4; they are similar in gene sequences and protein structures due to the same ancestral gene), neurotrophin-5 (NT-5), and neurotrophin-6 (NT-6) [84]. Other cytokines, including leukemia inhibitory factor (LIF), insulin-like growth factor-1 (IGF-1), transforming growth factor (TGF), epidermal growth factor (EGF), fibroblast growth factor (FGF), and platelet-derived growth factor (PDGF) [85], have also been detected in the CNS and reported to modulate neurogenesis, but they are not defined as NTs. Both neurons and neuroglial cells have been shown to synthesize and secrete NTs, which in turn act on all these cell types within the CNS.

Among NTs, BDNF is considered to exert the most significant and extensive neurotrophic effects; therefore, BDNF is also the most extensively studied NT in healthy and diseased brains [86–88]. The expression and maturation of BDNF protein is a multistage process. The original BDNF protein is synthesized in the endoplasmic reticulum and folded into pre-pro-BDNF [89]. After pre-pro-BDNF is translocated into the Golgi apparatus, the precursor signal sequence is cleaved to produce the pro-BDNF isoform [90]. Then, pro-BDNF is further cleaved to form mature BDNF (mBDNF), which has full physiological functions [91]. There has been substantial evidence indicating that BDNF is fundamental in the effectuation of LTP, mediating both functional and structural plasticity in the CNS, and BDNF has been discovered to modulate neuronal survival and neurogenesis in human hippocampus [92, 93]. Accordingly, patients with neurodegenerative diseases tend to have lower BDNF concentrations detected in their blood and brain [94, 95]. Some researchers hypothesized that since neuroinflammation is known to affect several BDNF-related signaling pathways, abnormal BDNF levels in diseased brains might be due to the chronic inflammatory state [96, 97]. The current study of BDNF’s effects is largely focused on the effects of antidepressant treatment on BDNF-mediated neuronal plasticity [98].

#### Neurotrophin receptor signaling pathways

BDNF signaling in receptor cells is generally categorized into receptor tyrosine kinase B (TrkB) pathways and the relatively low-affinity p75 neurotrophin receptor (p75NTR) pathways [96]. BDNF-mediated neuronal survival signaling can be summarized as follows [92]: released mature dimeric BDNF binds to presynaptic and postsynaptic TrkB in paracrine or autocrine manners, which results in the autophosphorylation and activation of TrkB. Consequently, several signaling pathways including phospholipase C-γ (PLC-γ), phosphoinositide 3-kinase (PI3K)-AKT, and extracellular signal-regulated kinase (ERK) are activated. First, BDNF-dependent PLC-γ activation can increase the Ca^2+^ neuronal response by activating downstream inositol triphosphate (IP3) or protein kinase C (PKC) cascades; meanwhile, the TrkB–PLC-γ–PKC axis decreases the inflammation-related apoptosis cascade by inhibiting glycogen synthase kinase-3β (GSK-3β). Second, the PI3K–AKT axis can induce de novo BDNF expression by activating mTOR-dependent BDNF translation [99, 100]. Third, BDNF can activate the transcription factor cyclic AMP response-binding protein (CREB) by inducing the ERK pathway, which regulates the expression of genes mediating neuronal survival, growth, and LTP. On the other hand, even though both pro-BDNF and mBDNF can bind p75NTR which may trigger nuclear factor-kappa B (NF-κB) signaling, its overall neurotrophic effects remain controversial and currently have no clear evidence to reach a conclusion [101].

During neuroinflammation, BDNF might regulate NF-κB-dependent proinflammatory gene expression through yet unclear mechanisms. Upregulated proinflammatory cytokines directly induce the production of various neurotoxic factors, such as reactive oxygen species (ROS), and more proinflammatory cytokines like IL-1β, IL-6, tumor necrosis factor-α (TNF-α), and chemokines [102, 103]. Mature BDNF is released into the synapse gaps from either presynaptic boutons or postsynaptic dendritic sites. Cis- or trans-activation of TrkB by BDNF plays different roles in synaptogenesis, Hebbian plasticity, and homeostatic plasticity. For example, TrkB activation at presynaptic sites contributes to presynaptic terminal formation whereas TrkB activation at postsynaptic sites promotes the formation of postsynaptic specializations [87].

#### Neurotrophin levels and neurogenesis during depression

In recent years, researchers have shown great interest in the measurement of peripheral BDNF levels associated with various mental diseases. Since significant reductions in serum BDNF levels were verified in both MDD animal models and patients [104, 105], it has been defined as a potential biomarker for depression assessment in some studies [106, 107]. For example, ameliorated depressive symptoms are in accordance with increased serum BDNF levels in MDD patients treated with effective antidepressants [108]. Treatment-resistant depression (TRD) patients tend to exhibit lower BDNF levels in corresponding brain structures such as the hippocampus [109]. Despite these facts, the time-course correlation between BDNF levels and antidepressant effects seems to be indirect: drugs and electroconvulsive therapies gradually increase serum BDNF levels in MDD patients, while their antidepressant effects appear quickly [110]. Moreover, it is noteworthy that the MDD-modulated BDNF levels were reported by Karege et al. to be initiated from reduced BDNF concentrations in the CNS rather than released from platelets [111]. Accordingly, synchronism between the release of BDNF in the CNS and serum has been recorded [112], while the relationship between serum BDNF levels and platelet-derived BDNF remains to be further investigated [113]. Also, the decreased serum BDNF levels measured in MDD patients were observed to synchronize with upregulation of the proinflammatory cytokines such as IL-6 and IL-8 [114, 115]. Altogether, neuroinflammation under chronically stressful conditions usually leads to reduced neurotrophin levels and thus inhibited neurogenesis during the course of depression, which can be partially mitigated with antidepressant treatments.

On the other hand, however, cell-to-cell communications within the CNS microenvironment could be more complicated. For instance, based on spatially patterned antibody microchips, the mediators of neuron–immune interactions were categorized by Deng et al. into 12 types of secretomes, including cytokines, NTs, and neuron-derived exosomes [116]. They also found that pairwise neuron–macrophage interactions and neuron–microglia interactions in AD models exerted distinct effects on modulating immune responses and neuron secretion [116], which highlighted the heterogeneity of secretion from single cells within the CNS under pathological conditions.

### Neuroinflammation attenuates neurogenesis

#### Microglial differentiation during brain innate immunity

Microglia are CNS-specific immune cells that affect the development and maintenance of the neural environment as well as the response and repair to injury [117]. Transmembrane protein 119 has been identified as the unique biomarker to distinguish microglia from peripheral monocyte-derived macrophages [118]. Similar to peripheral macrophages, microglia exhibit diverse phenotypes under different conditions to maintain homeostasis of the CNS through phenotypic transformation. For example, microglia can transdifferentiate into the M1 (proinflammatory) phenotype that upregulates the inflammatory response by expressing proinflammatory cytokines, or into the M2 (immunoregulatory) phenotype that inhibits inflammation and regulates tissue repair [119]. In vitro, cultured microglia have been shown to be induced by LPS or interferon-γ (IFN-γ) to differentiate to the M1 (proinflammatory) phenotype and to be induced by IL-4 or IL-13 to differentiate to the M2 (immunoregulatory) phenotype in previous studies [120]. In addition, microglia with the proinflammatory phenotype can enhance neuroinflammation responses within lesion areas through various mechanisms (see below), including recruiting peripheral monocytes and activating astrocytes [121, 122]. Various studies have shown that inhibiting the reactivity of microglia by reversing the differentiation direction leads to improved neurogenesis and cognitive performance in animal models by inhibiting neuroinflammation [123, 124]. However, it is noteworthy that summarizing the morphological and functional diversities of microglia into a binary opposition of M1 and M2 phenotypes by past studies is oversimplified. More recent studies revealed the indispensable roles of microglia in maintaining brain immune homeostasis and regulating neuronal activity under physiological conditions [125]. In addition, the M1 or M2 phenotype designation is not sufficient to summarize microglial functions under chronic stress [17]; for example, immunologically reactive microglia were found to mediate tissue damage and repair processes simultaneously [126, 127], which reflects the complexity of neuroimmune systems.

#### The proinflammatory roles of microglial cells in depression

Microglial differentiation into the proinflammatory phenotype has long been recognized to be secondary to neuronal injuries [17]. To date, several biomarkers for the proinflammatory phenotype have been identified [118], including Iba-1, CD11b, CD68, HLA-DR, etc. According to Farooq et al., CD11b expression is upregulated in MDD-related encephalic regions including the hippocampus and amygdala of CUMS mice [128]. Later, they also discovered that CD11b upregulation could be partially reversed by the administration of the antidepressant fluoxetine, which is consistent with the alleviation of CUMS-induced behavioral disorders [129]. Similarly, Preez et al. modified the CUMS model by adding a subsequent 6 weeks of social isolation (referred to as UCMSI). This procedure led to increased microglial proinflammatory differentiation and astrocytosis in the DG of the hippocampus, along with inhibited DG neurogenesis and several depressive-like behavioral changes in UCMSI animals [130]. These results uniformly suggest the critical role of microglial cells in mediating neuroinflammation during MDD progression (Fig. 1C; the corresponding experimental methods were described in Additional file 1). Moreover, abnormal levels of various bioactive elements within the CNS have also been shown to be involved in the regulation of microglia-mediated innate neuroinflammation. Mao et al. discovered the downregulation of miR-195 in the hippocampus during the chronic brain hypoperfusion process. miR-195 alters the communication between neurons and microglia by regulating CX3CL1–CX3CR1 signaling. miR-195 overexpression inhibited the chronic brain hypoperfusion-induced microglial differentiation to the proinflammatory phenotype, manifested by increased proportions of CD206^+^/Iba-1^+^ signals in hippocampal microglia [131]. Microglial proinflammatory differentiation is also the main cause of perioperative neurocognitive disorders. Luo et al. discovered through metabolomics that the glycolytic inhibitor 2-deoxy-d-glucose negatively regulates the proinflammatory differentiation of microglia and thereby inhibiting neuroinflammation in the hippocampus, alleviating the cognitive impairment caused by perioperative neurocognitive disorders, which is indicated by the decreased CD86^+^CD206^−^ ratio of microglia in the hippocampus [132].

Based on the effects of microglia described above, it is reasonable that some anti-inflammatory medicines, such as minocycline, have been reported to exert profound neuroprotective effects by diminishing microglial proinflammatory differentiation in the hippocampus, which is correlated with accelerated DG neurogenesis and better spatial memory under neuroinflammatory conditions [63, 133–135]. For example, in a rat model of cognitive impairment caused by sleep deprivation-induced neuroinflammation, the introduction of minocycline treatment was observed to improve the attenuated neurogenesis during sleep deprivation by inhibiting microglial proinflammatory differentiation and reducing the levels of microglia-released proinflammatory cytokines, which profoundly reduced the impairment of spatial memory [124]. As for the clinical application of minocycline, various trials have been conducted with depressed patients, in which minocycline showed promising potential to attenuate depressive symptoms in patients and improve their responses to antidepressants, even for TRD patients, while its long-term effects with large number of subjects remained ambiguous [136]. Taken together, microglial proinflammatory differentiation and the corresponding neuroinflammatory responses in the hippocampus during depression could be reversed by anti-inflammatory medicines, thereby facilitating hippocampal neurogenesis.

#### Microglial cells and P2X7-NLRP3 inflammasome signaling

Among different inflammasome cascades, the NLRP3 inflammasome signaling has received increasing attention in the study of brain innate immunity because of its relatively high expression in microglia [4]. Inside microglia, NLRP3 inflammasome activation was reported to be triggered by the purinergic P2X7 receptor bound to adenosine triphosphate (ATP), which induces potassium efflux to activate the downstream intracellular signaling [137]. Defined as “sterile inflammation” of the brain, this P2X7–NLRP3 inflammasome cascade in microglia is independent of the recognition of pathogen-associated molecular patterns [137, 138] and plays an important role in the pathophysiology of depression. In clinical observations, single nonsynonymous nucleotide polymorphisms in the human P2X7 receptor gene have been reported to be correlated with the risk of MDD aggravation in patients with affective mood disorders [139]. Furthermore, P2X7 receptor knockout in mice prevented the development of depressive-like symptoms under LPS treatment [140].

The NLRP3 inflammasome activates caspase-1, which subsequently cleaves pro-IL-1β and pro-IL-18 into their active, proinflammatory and releasable forms [141]. Under the steady state, IL-1β release induced by the microglia-related NLRP3 inflammasome was considered contributive to neuroprotection in excitotoxin-damaged mouse retinas [142]. However, IL-1β at high levels could be excitotoxic and hinder synaptic transmission [143]. For instance, hippocampal NLRP3 and IL-1β upregulation was detected in LPS-stimulated mice with depressive-like behaviors, and the subsequent ketamine treatment was found to exert the antidepressant effects via suppressing NLRP3 inflammasome signaling in the hippocampus [144]. Outside the CNS, the IL-1β upregulation induced by the NLRP3 inflammasome has been demonstrated to direct the differentiation of naïve CD4^+^ T cells into T helper 17 cells, which is known to promote the BBB permeability of peripheral immunocytes by weakening tight junctions, thus exacerbating neuroinflammation [145].

#### The neuroprotective functions implemented by microglial cells

Apart from mediating innate immunity in the CNS, increasingly extensive studies have revealed novel neuroprotective mechanisms implemented by highly specialized microglial subtypes under physiological conditions. Although microglia were classically viewed as being responsible for synaptic refinement and brain wiring in healthy brains [146, 147], not until recently have GABA-receptive microglial subtypes been discovered to selectively recognize and sculpt developing inhibitory circuits [148]. The dysfunction of this microglial subtype often leads to impaired synaptogenesis of GABAergic neurons and hyperexcitability [149, 150]. The activation of the complement (C3, C1q) pathway has long been considered an “eat me” signal to promote microglia-mediated identification of diseased synapses that need to be sculpted [151]. Meanwhile, certain “don’t eat me” counterparts, like CD47-related signaling, are required to balance the complement pathway [152] to avoid the excessive microglia-mediated phagocytosis of synapses, which plays an important role in synaptic loss and cortical functional connectivity disorders in depression [153]. In addition, the cytokine IL-33 derived from astrocytes and neurons was found to play a vital role in regulating microglial metabolic adaptation and synapse engulfment, which contributes to synapse remodeling, neural circuit development, and memory consolidation [147, 154, 155].

Moreover, Zhang et al. identified the Arg1^+^ microglial subtype located in the hippocampus, the proliferation, and maturation of which were driven by IL-4 signaling [156]. This Arg1^+^ microglia subtype is responsible for secreting additional BDNF which maintains microglial self-renewal, adult neurogenesis in the DG, and working memory under stress conditions [156, 157]. However, BDNF released from microglia was also reported to promote the transdifferentiation of astrocytes and microglia to release proinflammatory cytokines, causing aggravated neuroinflammation [158]. In conclusion, the neuroprotective effects exerted by microglia under both physiological and pathological conditions should not be neglected.

### Astrocytes participate in neuroinflammation

Astrocytes provide nutrients to neurons and contribute to temporal integration of neural activity by ensuring the functioning of synapses and driving action potentials [159]. They have also been illustrated to modulate neurological diseases in various manners. Similar to microglial cells, astrocytes could be induced into a reactive neurotoxic A1 phenotype in response to proinflammatory mediators and CNS injuries [160]. This process is defined as astrocytosis and is fundamental to cope with immune attack, chronic neurodegenerative disease, or acute trauma [161, 162]. McAlpine et al. reported that amyloid-β (Aβ) deposition during AD progression leads to the upregulation of IL-3 in astrocytes and IL-3 receptor α in microglia. Microglia sensitized by astrocyte-derived IL-3 clear Aβ aggregates, thus rendering IL-3 among crucial mediators of astrocyte–microglia crosstalk to resist AD progression and cognitive decline [163]. Yshii et al. discovered that astrocyte-specific IL-2 delivery in mice resulted in enhanced proliferation of reactive astrocytes and brain-resident Treg cells, which alleviated pathological neuroinflammation without affecting the peripheral immunity [164]. Moreover, various astrocyte-derived cytokines and metabolites have been found to exert neuroprotective effects and facilitate neuronal survival, including glutamate and TGF-β [165, 166]. In addition, proinflammatory microglia have been discovered to mediate the conversion of astrocytes into the A1 phenotype in various neurological diseases by secreting IL-1α, TNF-α, and C1q [167], while the blockade of this microglia-mediated conversion exhibited neuroprotective effects in Parkinson's disease (PD) models [168].

Researchers have also focused on the roles of astrocytes in facilitating the crosstalk between peripheral immunity and the CNS parenchyma due to the close contact between astrocytes and BBB components such as endothelial cells and pericytes [169]. Gimsa et al. reported that the reduced glutamate internalization of reactive astrocytes mitigated the production of the gap junction constituent connexin 43, thus increasing the permeability of the BBB to peripheral lymphocytes [170]. Moreover, bidirectional communications between reactive astrocytes and peripheral immunocytes have been revealed to modulate the recruitment of peripheral immunocytes to bypass the BBB and infiltrate into the CNS parenchyma. Astrocyte-derived C–C motif chemokine ligand 2 (CCL2) and C–X–C motif chemokine ligand 10 (CXCL10) were reported to induce extravasation and infiltration of peripheral macrophages, monocytes, T lymphocytes and promote their subsequent transdifferentiation into proinflammatory phenotypes [171–173]. Similarly, astrocyte-derived CXCL12 was shown to be responsible for the recruitment of pathogenic B lymphocytes [174]. On the other hand, astrocytes were also found to respond to inflammatory cues from infiltrated peripheral immunocytes. For example, IFN-γ produced by peripheral Th1 lymphocytes was reported to upregulate IFN-γ receptor 1 and MHC-II expression in astrocytes in the experimental autoimmune encephalitomyelitis (EAE) mouse models, which enables astrocytes to perform antigen-presenting functions [175]. IL-17 secreted from pathogenic Th17 lymphocytes was revealed to trigger proinflammatory transcriptional activities in astrocytes in EAE and multiple sclerosis (MS) models, according to single-cell RNA sequencing [176, 177].

### Oligodendrocytes participate in neuroinflammation

Oligodendrocytes were traditionally considered inactive in the process of neuroinflammation due to their highly differentiated and specialized nature for myelination [178]. However, various mechanisms of oligodendrocytes in CNS immunomodulation have been recently identified, which greatly improved the general understanding of the roles of oligodendrocytes in neuroinflammation [179–181]. It is noteworthy that many of these mechanisms depend on the intercommunication between oligodendrocytes and reactive astrocytes under pathological conditions [182]. For example, proinflammatory cytokines like TNF-α derived from A1 astrocytes have been found to initiate programmed death of adjacent oligodendrocytes, leading to mitigated remyelination and subsequent neuronal injuries [183, 184]. What’s more, the oligodendrocyte death and myelin loss might further induce adaptive autoimmune responses during the pathogenesis of MS [185]. On the other hand, certain inflammatory cues secreted from reactive astrocytes, such as CXCL1 and CCL2, also contribute to the mobilization and recruitment of oligodendrocyte progenitor cells (OPCs) to the inflammation sites [182, 186], which increases remyelination after the maturation of OPCs and helps the restoration of immunocompromised neural functions.

The diverse effects of oligodendrocyte–astrocyte communications might be attributed to the heterogeneity of reactive astrocytes. Oligodendrocytes have also been reported to be involved in CNS immunomodulation in astrocyte-independent manners. Papaneophytou et al. discovered that the downregulation of gap junction constituent connexin 47 in oligodendrocytes aggravates the disruption of blood–spinal cord barrier and the infiltration of peripheral immunocytes in MS models [187]. Similarly, aberrantly accumulated oligodendrocytes could interfere with astrocytes’ vascular interactions, disrupting the integrity of BBB in MS models [188]. In addition, diseased oligodendrocytes were found to secrete proinflammatory cytokines, including IL-1β, IL-6, and IL-17, which in turn modulate the functions of other neuroglial cells at inflammation sites [182, 189].

### Neuroinflammation augmented by the aberrance of peripheral immunocytes attenuates neurogenesis

#### Interactions between peripheral immunity and MDD

The existence of peripheral immune cells within the CNS used to be considered negligible under normal physiological conditions. Anatomically, the brain and spinal cord are surrounded by the meninges, which are richly vascularized and innervated multilayer structures consisting of the outer dura mater, the middle arachnoid, and the inner pia mater [190]. CSF produced by choroid plexus epithelial cells flows both within the meninges [191] and into cervical lymph nodes [192], thus facilitating the communication between the meninges and peripheral lymphatic system. Therefore, when neuroinflammation occurs, peripheral immunocytes can respond to antigens presented in the CNS, selectively penetrate the BBB in different ways into the meninges or other cerebral immune-privileged sites [193, 194], and then contribute to sustained immune responses for both autoimmunity and infectious diseases of the CNS [195].

#### Blood leukocytes in MDD

The studies of immune cell trafficking between the meninges and peripheral lymphatic system have mainly focused on peripheral T lymphocytes [196, 197]. CD8^+^ T cells recognize linear epitopes presented by the MHC class I molecules expressed by almost all cells and differentiate into cytotoxic T lymphocytes to kill infected cells or tumor cells [198]. CD4^+^ T lymphocytes are composed of various subtypes according to their phenotypes, including helper T1 (Th1), Th2, Th17, and regulatory T (Treg) cells [199]. Treg cells can be further divided into thymus-derived Treg cells (tTreg cells) directly developed from CD4-single positive (CD4-SP) thymocytes or peripheral-derived Treg cells (pTreg cells) differentiated from naïve CD4^+^ T cells upon TCR ligation with TGF-β and IL-2 [200]. Other Th cells also differentiate from naïve CD4^+^ T cells upon TCR ligation, although with different cytokine requirements [201].

In MDD patients, alterations in peripheral immune cells at the systemic level have been frequently reported. Blood T cells in MDD patients exhibit reduced mitochondrial respiration and glycolytic capacity [202]. Furthermore, there is a link between the suicidality of MDD patients and premature Th cell aging and increased proportions of Th17 cells in the blood [200]. Also, a trend of lower blood CD8^+^ T cell numbers and lower CD4^+^/CD8^+^ T cell ratios is associated with MDD severity [203]. In addition, reduced Treg frequency or impaired Treg function has been observed in the blood of MDD patients [204–206]. These findings suggest that T cells are involved in MDD progression.

In MDD state, profound changes occur to blood leukocytes other than T cells. MDD patients exhibit higher neutrophil/lymphocyte and monocyte/lymphocyte ratios [207, 208]. Accordingly, increased numbers of proinflammatory monocytes have been observed in the blood of MDD patients [203, 209]. Gene expression analysis suggests mitochondrial dysfunction, premature aging, and high expression of proinflammatory cytokines [210]. Although the changes in total B cell numbers are not consistent in different studies [211], blood CD1d^+^CD5^+^ and CD24^+^CD38^hi^ transitional B cells, which show immune-regulatory functions, are reduced in MDD patients [212]. Furthermore, blood B cells in MDD patients show shorter telomeres as well as T cells, indicating the accelerated loss of B-cell functions [213]. There are also reports of reduced numbers of CD56^+^CD16^–^ regulatory NK cells in MDD patients [214].

#### The discovery of brain-resident leukocytes

The rediscovery of fluid exchange between the CSF and interstitial fluid of the brain could serve as a sufficient delivery mechanism for soluble factors released from meningeal leukocytes to modulate neurological diseases [215]. The origin of meningeal leukocytes has been intensely investigated [216–220]. It has been established that most meningeal myeloid cells and B cells are not blood-derived. As skull-to-dura channels exist, it is speculated that meningeal leukocytes transit from the skull through these specialized channels [216, 217, 220]. However, Schafflick et al. questioned this possibility: they labeled skull leukocytes and the fluorescence signal lasted up to 17 days but dura leukocytes remained unlabeled throughput the process [218]. Niu et al. proposed another possibility by identification of hematopoietic stem cells residing in the meninges of adult mice at steady state [219]. There are also various resident peripheral leukocytes in the CSF and parenchyma [20, 21, 216]. With a tissue-specific expression profile, meningeal hematopoietic stem cells can provide the CSF and parenchyma with a constant supply of leukocytes more adapted to the local microenvironment [219]. Since dura myeloid subsets showed similar changes to their femur bone marrow counterparts in a mouse CUMS model [221], it is possible that the aberrance in blood leukocytes of MDD patients also occurs in brain-resident leukocytes. Future studies are required to address this issue.

#### CD4^+^ T lymphocytes and hippocampal neurogenesis

It has now been established that T lymphocytes contribute to brain development and immune homeostasis under physiological conditions. In 2009, Wolf et al. [222] found that systemic clearance of CD4^+^ T cells in the brains of mice led to reduced neurogenesis in the hippocampus, impaired spatial memory indicated by the MWM test, and diminished BDNF expression. The clearance of CD8^+^ or B cells in the brain, however, did not inflict similar effects on neurogenesis [222]. Such a role has been confirmed by several studies. For example, Negrini et al. reported that BALB/c nude mice show social disorders, accompanied by decreased expression of IFN-γ in the prefrontal cortex and the absence of T cells in the meninges [223]. The absence of a residential CD4^+^CD69^+^ T lymphocyte subset in healthy murine brains resulted in suspended microglial activation, together with developmental defects of synapses and behavioral abnormalities [224]. Furthermore, Treg cells were identified to protect female mice from colony stimulating factor 1 (CSF1) intrathecal administration-induced neuropathic pain by suppressing microglial differentiation into proinflammtory phenotypes [225]. Together, these studies indicate protective roles of CD4^+^ T cells in maintaining immune homeostasis.

#### Cytokines for brain-resident T lymphocytes and hippocampal neurogenesis

Various cytokines in the CNS have been shown to affect the differentiation and functions of brain-resident T lymphocytes [226, 227]. For example, astrocyte-targeted IL-2 expression expanded brain-resident Treg cells under pathological conditions without impacting the peripheral immunity [164]. Moreover, exercise was found to induce the upregulation of CD4^+^CD25^+^ Treg cells and anti-inflammatory responses [228]. Derecki et al. reported that the accumulation of CD4^+^ T lymphocytes in the meninges of a mouse model after MWM training was in accordance with enhanced visuo-spatial memory. According to their results, the numbers of meningeal CD4^+^ T cells expressing CD69 increased remarkably after training, while meningeal CD8^+^ T cells did not exhibit the induction of the early activation marker CD69. Further detection showed that the proportion of CD4^+^CD25^+^Foxp3^+^ Treg subsets in meningeal cells of MWM-trained mice was elevated, accompanied by increased IL-4 secretion. Meanwhile, the subpopulations of myeloid cells in the meninges were also confirmed to respond. All these changes were correlated with improved visuo-spatial memory, suggesting they contribute to hippocampal neurogenesis [229].

For brain-resident CD4^+^ T lymphocytes, the CD4 coreceptor ligand IL-16 controls their trafficking and biological characteristics. IL-16 was originally identified as a T-cell chemoattractant in the 1980s. Subsequent researches have mainly focused on its roles in inflammation regulation [230]. In particular, Treg-associated IL-16 functions include accelerating the proliferation of CD4^+^CD25^+^ T lymphocytes, de novo induction of Foxp3 and migration of Foxp3^+^ T lymphocytes in the case of long-term culture with IL-2, the key factor of Treg differentiation and proliferation [231]. IL-16 also modulates neuronal excitability and synaptic activity in the mouse hippocampus ex vivo, serving as a neuroprotective factor against excitotoxicity [232, 233]. Notably, in the steady state, hippocampal and cerebellar granule neurons produce and secret considerable amounts of secretory neuronal interleukin-16 (NIL-16), a splice variant of IL-16, into the systemic circulation [234, 235]. NIL-16 is a cytoplasmic protein detected only in neurons in the cerebellum and hippocampus [236]. The N-terminus of NIL-16 selectively interacts with various neuronal ion channels, which is similar to the function of many other PDZ domain proteins as intracellular scaffold proteins [237]. The C-terminus of IL-16/NIL-16 can be recognized and cleaved by caspase-3, resulting in the release of secretory IL-16/NIL-16 [238]. The expression of the IL-16 receptor, namely the CD4 protein, also exists in granule neurons. These neurons treated with NIL-16 induced the expression of c-fos through the tyrosine phosphorylation signaling pathway, which indicates that NIL-16 could function in an autocrine manner. Conclusively, NIL-16 is a protein with dual functions in the nervous system, acting as both a secreted signaling molecule and a scaffold protein [236]. NIL-16-induced inhibition of neuroinflammation contributes to hippocampal neurogenesis, while promoted neurogenesis might in turn further elevate NIL-16 secretion.

Meanwhile, it is noteworthy that the effects of immune-regulatory cytokines during neuroinflammation could be source-specific and context-dependent [239]. By diverting IL-2 production, Whyte et al. identified the different secretory manners of IL-2 released from Treg cells, CD8^+^ T lymphocytes, and NK cells (autocrine or paracrine), as well as different downstream functional mechanisms [240]. Therefore, targeting cytokine production in specific cell types could be conducive to overcoming the adverse effects of anti-inflammatory treatment.

## The effects of neuroinflammation on memory impairment via modulating the hypothalamic–pituitary–adrenal (HPA) axis

### Abnormalities in the HPA axis during depression

The HPA axis has been known as one of the canonical stress hormone systems functioning in response to external pressures [241]. In both mice and humans, the corticotropin-releasing hormone (CRH) secreted from paraventricular neurons in the hypothalamus binds and activates the CRH receptor in cells of the pituitary. The above process causes adrenocorticotropic hormone (ACTH) secretion into peripheral circulation. Then, ACTH transported to adrenal glands induces the release of glucocorticoids (primarily corticosterone in mice and cortisol in humans) [242]. Glucocorticoids have been reported to act on nearly all parts of the body and function in various physiological processes by activating glucocorticoid receptors (GRs), exerting anti-inflammatory and immunosuppressive effects and regulating neuronal functions of the limbic system (including the hippocampus) [243, 244]. However, upregulated glucocorticoids also bind to GRs located in multiple brain regions including the hypothalamus and the pituitary, maintaining the homeostasis of the HPA axis through negative feedback loops [245].

The dysregulation and imbalance of the HPA axis have been observed in a prominent proportion of MDD clinical cases and are believed to be initiated from the malfunction of GR-dependent negative feedback of the HPA axis, which is defined as GR resistance [246, 247]. Meanwhile, the alleviation of GR resistance was discovered in MDD patients after receiving effective antidepressive therapies [248]. Genetic variations in the GR-encoding gene *NR3C1* and mineralocorticoid receptor (MR)-encoding gene *NR3C2* have been manifested in both bioinformatics and clinical studies to correlate with increased risks of cognitive impairments and HPA axis dysregulation [246, 249]. In specific, aberrant glucocorticoid levels have been found to participate in regulating hippocampal neuroinflammation [250, 251], and to interfere with the development, differentiation, and function of T lymphocytes through TCR signaling [252, 253]. On the other hand, certain inflammatory cytokines have been reported to stimulate HPA axis activity and glucocorticoid secretion, including IL-1, IL-6, IL-10, and TNF-α [254]. In addition, various genetic mouse models of MDD have been constructed by selective breeding. In these models, aberrant corticosterone levels were found to be correlated with the severity of depressive-like behaviors [255]. In conclusion, HPA axis dysregulation and GR resistance under chronic stress partially account for the exaggerated neuroinflammation observed during depression, in which glucocorticoids play the central role in exerting anti-inflammatory effects [256].

### Hypercortisolism and neuroinflammation during depression

Nevertheless, GR resistance initiated from an imbalanced HPA axis also synchronizes with glucocorticoid hypersecretion observed in MDD patients, which was defined as hypercortisolism [246]. This prompted us to explore the potential explanation based on our understanding of the role of neuroinflammation in accelerating MDD progression and cognitive impairments [257]. To date, several possible mechanisms have been proposed, which have mainly focused on glucocorticoid-induced upregulation of proinflammatory genes. Toll-like receptor families (TLRs) are claimed to be upregulated and overactivated under hypercortisolism through multiple mechanisms, which subsequently augment the secretion of TNF-α [258]. Bullsilo et al. showed that glucocorticoids induce NLRP3 upregulation at both the mRNA and protein levels, and the NLRP3 inflammasome sensitizes the release of IL-1β, TNF-α, and IL-6 from mature macrophages [259]. Treatment with glucocorticoids and TNF-α synergistically increased the expression of the proinflammatory gene serpinA3 in vitro and in vivo [260].

Furthermore, persistent activation of the HPA axis and hypercortisolism during depression have been clarified to impair the immune surveillance mediated by peripheral lymphocytes [261]. Age-dependent dysregulation of the HPA axis leads to a decline in the peripheral pool of T lymphocytes in the thymus, since the signaling of hormone, neurotransmitter, and neuropeptide receptors expressed in thymocytes affects their maturation and function [262]. In addition, the effects of other brain-derived hormones and neurotransmitters on peripheral leukocytes have also been discovered; for instance, norepinephrine could bind to the β2 adrenergic receptors expressed on peripheral CD4^+^ T cells and B cells to regulate the corresponding immune responses [263]. Cytokines derived from peripheral leukocytes were also verified to bypass the BBB and modulate neuronal functions. Thus, the CNS and the peripheral immune system form bidirectional communications to promote the pathogenesis and progression of depression [264].

### Serotonin dysfunctions and neuroinflammation during depression

Serotonin/5-hydroxytryptamine (5-HT) is an important neurotransmitter synthesized from tryptophan, and serotonin metabolism has been observed to be reduced during depression [265]. Decreased cerebral serotonin levels in MDD patients could be reversed with the treatment of antidepressants [266]. The current hypothesis is that the catabolism of serotonin into kynurenine is highly related to neuroinflammation. The metabolites of kynurenine could be either “excitotoxic” quinolinic acid or “neuroprotective” kynurenic acid, which are mainly synthetized by microglia and astrocytes within the CNS, respectively [267, 268]. The serotonin/kynurenine ratio in the plasma and CSF, which indicates the activation of the kynurenine pathway, has been found to correlate with MDD occurrence and the intensity of depressive symptoms, according to both animal models and clinical data [269–271]. Also, since microglial kynurenine mono-oxygenase is responsible for catabolizing the synthesis of quinolinic acid from kynurenine, a mono-oxygenase-knockout mouse model was constructed, which verified the irreplaceable role of mono-oxygenase in stirring the kynurenine pathway toward excitotoxic effects under the treatment of inflammatory inducers such as LPS [272].

Moreover, cross-interactions between the serotonin system and HPA axis have been elucidated. Serotonin binds 5-HT1A receptors in hippocampal NPCs and regulates their reactivities to glucocorticoids [273], while the sensitivity of serotonin to bind to the serotonin transporter could be markedly influenced by endocrine regulation of the HPA axis [274].

## Conclusion

**Fig. 2:**
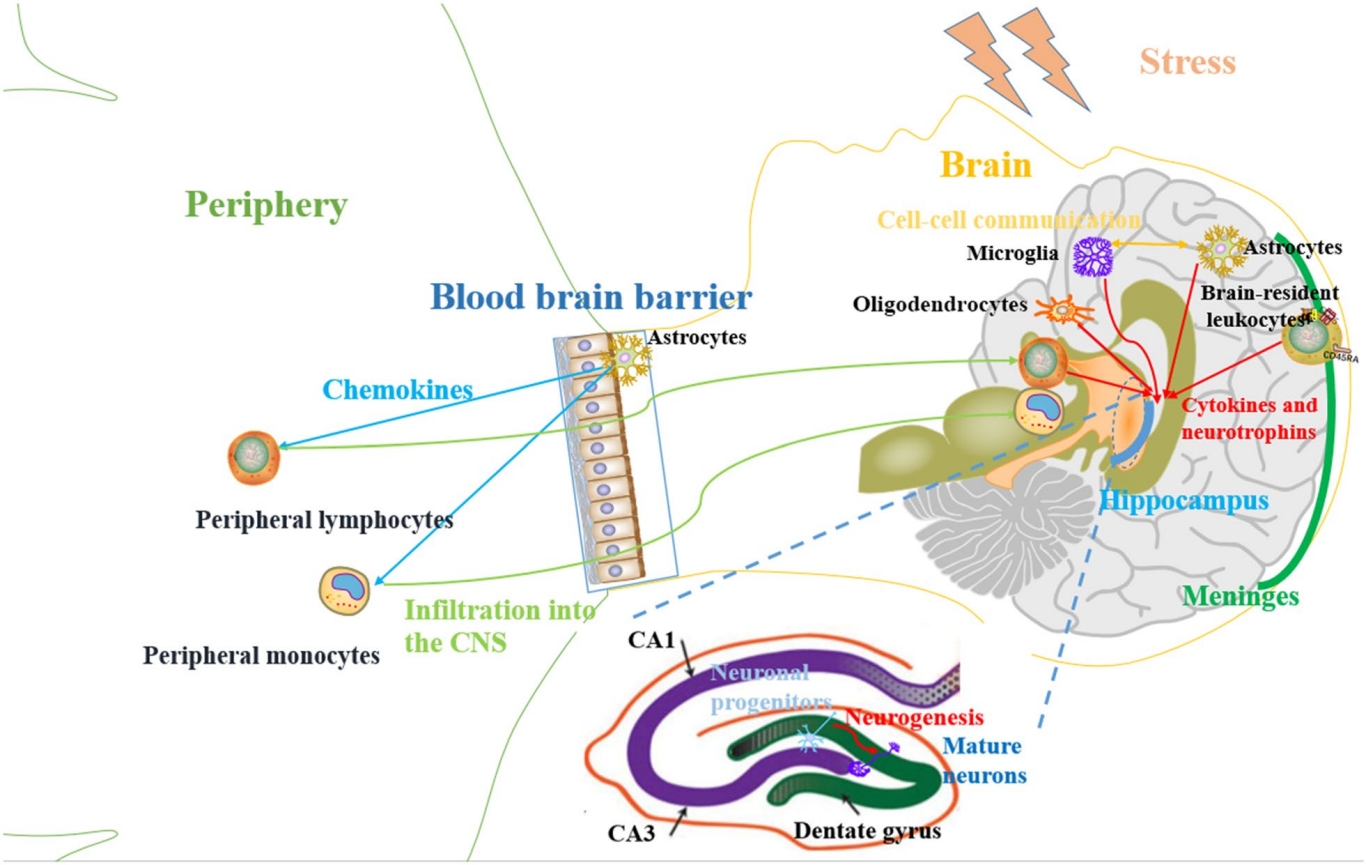
Schematic summary of the cell types that participate in the CNS immunoregulation and impact on memory functions by modulating hippocampal neurogenesis in the DG during neuroinflammation. As presented in this figure, under pathological conditions, the differentiation of microglia, astrocytes, and other neuroglia into reactive phenotypes modulates the CNS neuroinflammation and affects the DG neurogenesis. Brain-resident leukocytes in the meninges have been reported to influence the DG neurogenesis and memory functions. In addition, more blood immune cells bypass the BBB and infiltrate into the CNS. The intercommunication between these cell types, as well as neurons, greatly relies on various cytokines and neurotrophins

As reviewed above, the interplay between neuroinflammation and depression has been extensively explored in both preclinical and clinical studies. The impairments of the hippocampal structures and functions implemented by neuroinflammation during MDD progression correspond to attenuated memory performance. The current understanding could be summarized as follows: neurogenesis in the hippocampal dentate gyrus, the HPA axis, and the brain serotonin system are all closely related to the performance of multiple cognitive functions. In addition, the neuronal morphology, neural plasticity, and phenotypes of neuroglial cells within the same encephalic region also affect cognitive capabilities. Therefore, when neuroinflammation occurs in the course of depression, these factors are negatively regulated by excessive chronic neuroinflammatory responses, causing clinically observed cognitive impairments. Reactive microglia and astrocytes and peripheral proinflammatory cells that specifically penetrate the BBB into the CNS after receiving signaling molecules are involved in the mediation of neuroinflammation. By contrast, regulatory immune cells inhibit the neuroimmune responses after receiving specific stimulation, thereby protecting cognitive functions from damage (Fig. 2). The synthesis, secretion, and signal transduction of various types of secretory factors are indispensable in the effectuation of the cross-talks between abovementioned cell types (Table 1). All these mechanisms are supported by evidence from either clinical studies or animal models related to major depression, but more details still need to be further clarified, so as to achieve potentially better clinical therapeutic effects for the development of the corresponding treatments.

**Table 1:** Secretory factors reported in the study of emotional disorders and neuroinflammation

Secretory factor	Major producer cells in the brain	Major recipient cells in the brain	Biological effects in neuroinflammation	References
BDNF	Neurons; neuroglial cells	Neurons	Promote neurogenesis; maintain neuroplasticity and LTP; enhance neural survival	[83, 84, 92–94]
BDNF	Neurons; neuroglial cells	Neuroglial cells	Induce de novo BDNF expression; transcriptionally regulate the expression of cytokines	[99–103]
IFN-γ	NK cells; T cells	Microglia; astrocytes	Induce microglial proinflammatory differentiation; enable astrocytes to perform antigen-presenting functions	[120, 175]
IL-1β	Microglia; diseased oligodendrocytes	Various cell types	Be neuroprotective at physiological levels; be excitotoxic and hinder synaptic transmission at high levels; promote neuroinflammation; direct the differentiation of naïve CD4^+^ T cells into Th17 cells; stimulate HPA axis activity and glucocorticoid secretion	[141, 142, 189, 254]
IL-2	T cells; NK cells	T cells	Alleviated pathological neuroinflammation by promoting Treg proliferation; Promote the differentiation of naïve CD4^+^ T cells into Treg cells	[164, 200, 231, 240]
IL-4	Th2 cells	Microglia	Induce microglial immunoregulatory differentiation; promote hippocampal neurogenesis by accelerating the proliferation of Arg1^+^ microglia; upregulate BDNF secretion	[120, 156, 229]
IL-6	Microglia; diseased oligodendrocytes	Various cell types	Promote neuroinflammation; stimulate HPA axis activity and glucocorticoid secretion	[102–104, 189, 254]
IL-10	Treg cells	Various cell types	Restrain microglia/macrophage-mediated neuroinflammation; stimulate HPA axis activity and glucocorticoid secretion	[227, 254]
IL-13	Various cell types	Microglia	Induce microglial immunoregulatory differentiation	[120]
IL-17	Th17 cells; diseased oligodendrocytes	Various neuroglial cell types	Promote neuroinflammation by inducing proinflammatory transcriptional activities in neuroglial cells	[176–182]
IL-18	Microglia	Various cell types	Promote neuroinflammation after been activated by inflammasome signaling	[141]
IL-21	High affinity CD4^+^T cells	CD8^+^ T cells	Modulate the differentiation of brain-resident CD8^+^ T cells during infection	[226]
IL-33	Astrocytes; neurons	Microglia	Maintain microglial metabolic adaptation and phagocytic function; promotes microglial synapse engulfment and neural circuit development	[147, 154, 155]
IL-16	Treg cells	CD4^+^T cells; neurons	Accelerate the proliferation and differentiation of Treg cells; modulates neuronal excitability and synaptic activity; Inhibit neuroinflammation	[230, 231]
NIL-16	Certain neuronal types	CD4^+^T cells; neurons	Inhibit neuroinflammation; serve as a scaffold protein	[235–238]
TNF-α	Microglia; astrocytes	Various cell types	Be neurotoxic; promote neuroinflammation; induce cellular apoptosis and necroptosis; stimulate HPA axis activity and glucocorticoid secretion	[103, 104, 185, 186, 257]
CCL2	Reactive astrocytes	Various cell types	Induce extravasation and infiltration of peripheral macrophages, monocytes, T lymphocytes and their subsequent transdifferentiation into proinflammatory phenotypes	[171, 173, 182, 186]
CXCL1	Reactive astrocytes	Oligodendrocyte progenitor cells	Promote the mobilization and recruitment of oligodendrocyte progenitor cells (OPCs) to the inflammation sites	[182, 186]
CXCL10	Reactive astrocytes	Various cell types	Induce extravasation and infiltration of peripheral macrophages, monocytes, T lymphocytes and their subsequent transdifferentiation into proinflammatory phenotypes	[171, 172]
CXCL12	Reactive astrocytes	B lymphocytes	Induce the recruitment of pathogenic B lymphocytes into the CNS	[174]
Glucocorticoids	Adrenal cortical cells	Various cell types	Modulate hippocampal neuroinflammation; interfere the differentiation and function of T lymphocytes; induce the secretion of proinflammatory cytokines from peripheral immunocytes; activate inflammasome signaling	[249–260]
Serotonin	Certain neuronal types	Various cell types	Modulate neuroinflammation by its metabolisms in microglia; regulate the sensitivities of hippocampal neural progenitor cells (NPCs) to glucocorticoids	[267–274]

### Supplementary Information

**Additional file 1.** The materials and methods corresponding to the results presented in Fig. 1 are described Additional file.

## Data Availability

Not applicable.
