# Supplementary material for: Neuroinflammation, memory, and depression: new approaches to hippocampal neurogenesis
Source: J Neuroinflammation. 2023 Nov 27;20:283. doi: 10.1186/s12974-023-02964-x (PMC10683283; doi:10.1186/s12974-023-02964-x)
Supplement: Supplementary file 1 — Additional file 1. The materials and methods corresponding to the results presented in Fig. 1 are described Additional file. [file 12974_2023_2964_MOESM1_ESM.docx]

**Additional Materials**

The materials and methods corresponding to the results presented in **Figure 1.** are described as follows:

**1. Animal procedures**

All animal procedures were designed and performed according to the regulations of the Institutional Animal Care and Use Ethics Committee of the Beijing Institute of Basic Medical Sciences under the ethics approval number AMMS 2021-1356. In this study, mice were subjected to procedures modified from a previously reported chronic unpredictable mild stress (CUMS) model [1]. In brief, 8-week-old C57 BL/6 N male mice (provided by Beijing Vital River Laboratory Animal Technology Co., Ltd.) were maintained under specific pathogen-free conditions with controlled temperature, humidity and light-dark cycles for 7 days of adaptive feeding. Then, the mice were designated to receive various, erratic mild stresses for 8 consecutive weeks (as in **Table S1.**). After 8 weeks, CUMS-treated mice were subjected to the forced swimming test (FST) [2] and tail suspension test (TST) [3] to evaluate the development of major depressive disorder. Mice with major depressive disorder, together with negative controls without receiving CUMS procedures were sacrificed to obtain brains for the following assays. Each experimental group had at least nine surviving mice for sampling.

**2. Immunohistofluorescence (IHF)**

For tissue sections, mice were anesthetized, followed by thoracotomy and intracardiac perfusion with precooled saline and 2% PFA successively. Then, the whole brains were isolated, fixed with 4% PFA for an additional 24 hours and dehydrated with a graded series of sucrose solutions. After that, brain samples were embedded and sliced by a Dakewe CT520 cryostat microtome with a section thickness of 8 μm. Coronal sections of the hippocampus were stored at -80°C before staining procedures. For staining, tissue sections were treated with 0.3% Triton X-100 in PBS for 10 min, blocked with 10% donkey serum for 30 min, incubated with primary antibodies O/N at 4℃ and then with corresponding fluorescence-conjugated secondary antibodies at RT for 2 hours. Afterwards, 4',6-diamidino-2-phenylindole (DAPI, FluoroPure™ grade, Invitrogen, D21490) was applied to stain the nuclei, and the samples were mounted onto glass slides and imaged by an Olympus IX53 fluorescence microscope. The antibodies used are listed in **Table S2.**

**3. Statistical analysis**

The data presented were collected from three independent, parallel experiments and presented as the mean ± SEM. Statistical analysis was conducted using nonparametric tests (Mann-Whitney test), with GraphPad Prism software V8.2.1. p values < 0.05 were considered statistically significant.

**Table S1. Arrangement of mild stress during the CUMS procedure**

**Table S2. Antibodies for assays**

| **Antibody** | **Vendor and Cat. No.** | **Application** |
| --- | --- | --- |
| rabbit anti-Dcx | Cell Signaling Technology, 4604S | IHF (1:500) |
| rabbit anti-Iba1 | Wako, 019-19741 | IHF (1:200) |
| mouse anti-NeuN | Proteintech, 66836-1-AP | IHF (1:400) |
| goat anti-mouse IgG-  Alexa Fluor 488 | Abcam, ab150113 | IHF (1:500) |
| goat anti-rabbit IgG-  Alexa Fluor Plus 555 | Abcam, ab150078 | IHF (1:500) |
| goat anti-rabbit IgG  -Alexa Fluor Plus 488 | Abcam, ab150077 | IHF (1:500) |

**Reference**

[1] Burstein, O. et al. (2018) The Unpredictable Chronic Mild Stress Protocol for Inducing Anhedonia in Mice. *J Vis Exp*. (140):58184.

[2] Yankelevitch-Yahav, R. et al. (2015) The forced swim test as a model of depressive-like behavior. *J Vis Exp*. (97):52587.

[3] Can, A. et al. (2012) The tail suspension test. *J Vis Exp.* (59): e3769.
